# Supplementary material for: HANDSON Hand: Strategies and Approaches for Competitive Success at CYBATHLON 2024
Source: Bioengineering (Basel). 2025 Feb 24;12(3):228. doi: 10.3390/bioengineering12030228 (PMC11939478; doi:10.3390/bioengineering12030228)
Supplement: Supplementary file 1 [file bioengineering-12-00228-s001.zip › S2. Race_and_Rules.pdf]

7 Arm Prosthesis Race

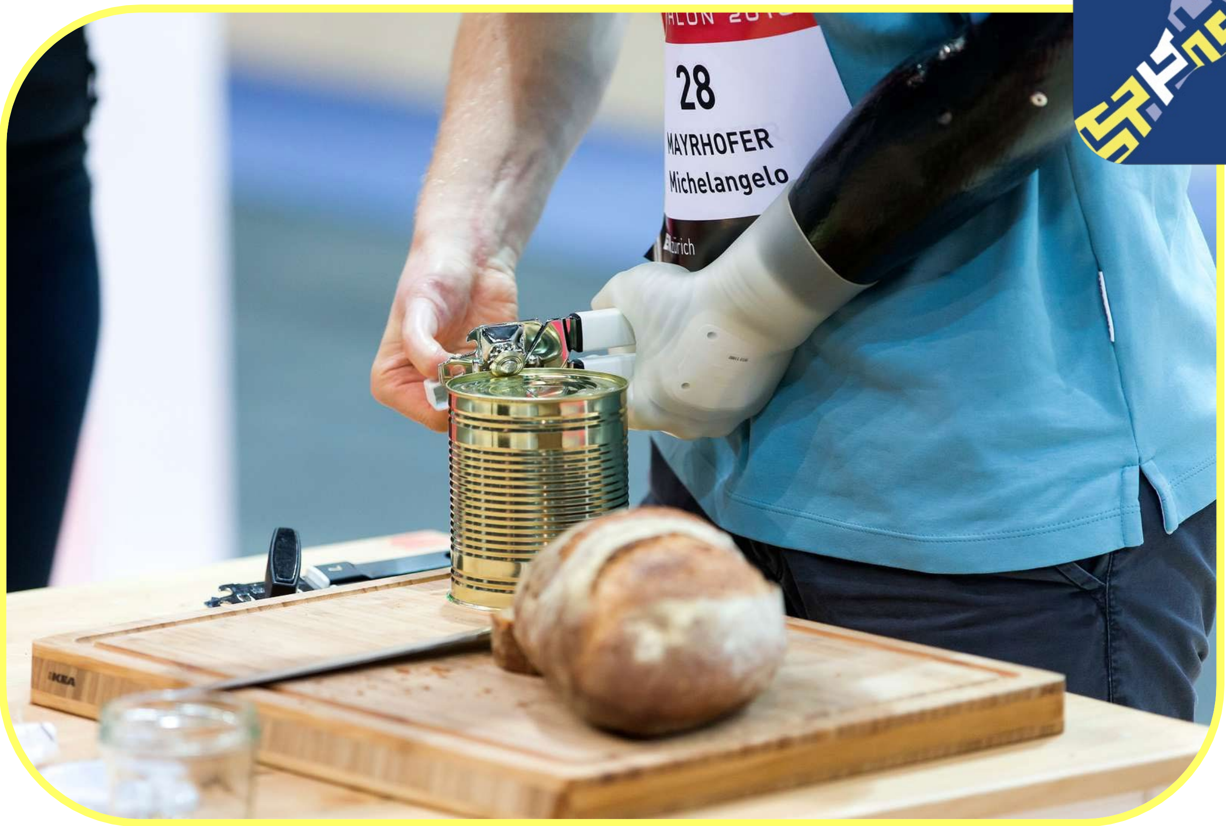

ARM pilot solving the Breakfast task during CYBATHLON 2016.

7.1 Introduction

A limb difference at the level of the forearm or above (e.g., due to an amputation or a congenital disorder) may lead to significant challenges when interacting with the physical environment. While many of the latest anthropomorphic hand prostheses provide a wide variety of grip patterns, their use and range of functions is often not fully satisfying for their users. The devices still lack some of the fundamental functionalities of a human hand such as wrist flexion and extension or the control of individual fingers. Missing degrees of freedom often result in non-physiological compensatory movements. Most devices do also not provide proprioceptive and haptic sensory information to their user which can lead to a lack of embodiment and acceptance of the prosthesis. Furthermore, the control of a hand prosthesis often requires significant cognitive and visual attention from their users. Due to these functional shortcomings many arm prostheses users abandon their device in the long run.

Arm prostheses which fulfil the users' expectations and needs have the potential to prevent device rejection. Additionally, prostheses that enable the functions of a human hand in a natural way may prevent secondary negative long-term effects due to non-physiological movements or anatomical asymmetry.

7.2 Eligibility criteria

In addition to the General Rules outlined in Chapter 4, the following specific rules apply for the Arm Prosthesis Race:

7.2.1 Pilots

In addition to the general pilot eligibility criteria set forth in chapter 4.5.1, pilots must fulfil the following criteria to be eligible for participation in the ARM race:

ARM-PIL-1 Pilots must have a transradial or more proximal amputation or dysmelia of at least one arm.

Comment 1 on ARM-PIL-1: The pilot should not have any residual function in the wrist.

Comment 2 on ARM-PIL-1: Please mind the following x-rays to check if your pilot candidate is eligible for the competition.

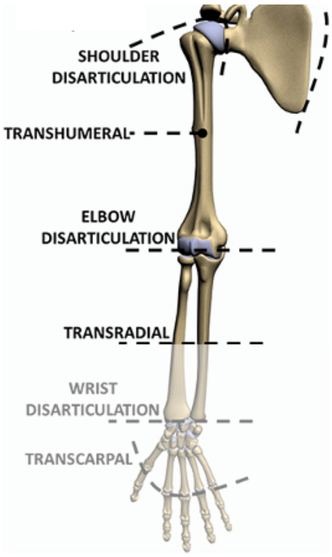

Eligible for the competition:
Transradial or more proximal amputation.

Not eligible for the competition:
Residual wrist structure and function.

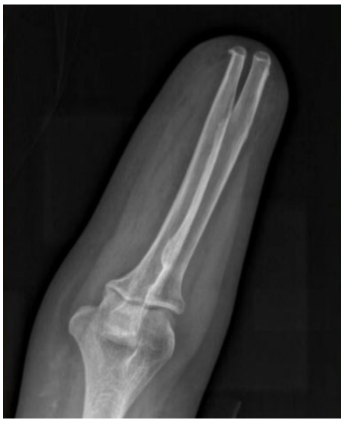

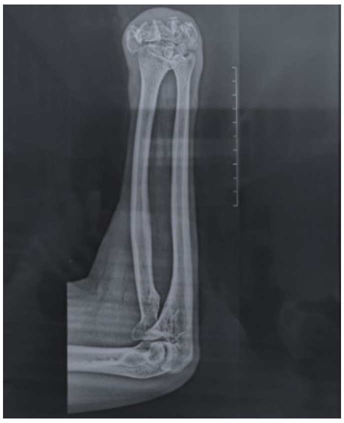

7.2.2 Technology

There are no specific eligibility criteria in addition to the general technology eligibility criteria set forth in Chapter 4.5.2.

General comments on the prostheses

- Unpowered or powered (motor or body powered) prostheses are allowed.
- The prosthetic device may have any number of actively driven (powered) joints (e.g., for hand opening/closing or wrist pronation/supination). The prosthetic device can have several passive or mechanically coupled joints (e.g., at the fingers). Body powered (e.g., cable driven) systems are also allowed.
- If not otherwise specified in the task specific rules, any technical modality (and their combination) to collect information about the environment is eligible (e.g., LIDAR, vision, ultrasound).
- There is no weight limit for the prosthesis.

7.3 Specific race rules

ARM-1 It is not allowed to use items such as trailers, backpacks, bags, pockets, ropes, or their clothes to carry objects of the racetrack (e.g., tools, plates, and bags of the tasks), but it is allowed to use such aids to carry components of the device (e.g., batteries, control units, replacement equipment, etc.).

ARM-2 It is not allowed to touch, i.e., get in contact with, any part of theprosthesis , while it is in direct contact with any blue part of an object.

Comment 1 on ARM-2: Part of the prosthesis includes any wired or wireless interfaces which can be used to control the function of the prosthetic arm (e.g., buttons, remote control units, user interface).

Comment 2 on ARM-2: Non-robust control of prosthetic hand function during postural changes of the arm is an issue for many arm prostheses users. They oftentimes resort to turning off the device to maintain a secure grip while carrying objects. While pragmatic and simple to implement, this approach is not satisfying to many users. Rule ARM-2 aims to encourage teams to seek novel solutions to device design and control to maintain robust function during postural changes of the arm.

ARM-3 Blue parts are only allowed to be manipulated or touched with the prosthetic hand (not including wrist, lower or upper arm).

ARM-4 It is allowed to touch several blue objects at a time.

ARM-5 If a pilot uses two prostheses, only one prosthesis is allowed to touch blue objects at a time.

Comment on ARM-5: If a pilot uses two prostheses, the pilot can decide for each manipulation which of the two prosthesis acts as the “prosthetic hand” and which one as their “non-prosthetic hand”. Note that handing a blue object from one prosthetic hand to another prosthetic hand is not allowed.

7.4 Task definitions

Each task is described in the following sections. If not otherwise defined, the direction of the race is (bottom) left to (top) right in all following figures.

7.4.1 Carry Bottles

7.4.1.1 Introduction

Forces that act on the prosthesis during the manipulation of heavy objects can cause a displacement of the prosthetic socket relative to the arm stump. In consequence the control signals recorded by traditional surface EMG can become unreliable leading to unsatisfactory function of the prosthesis.

In this task a series of bottles of different weights must be placed in a bottle crate and the crate must then be carried to a shelf, removed from the crate and placed on top of it.

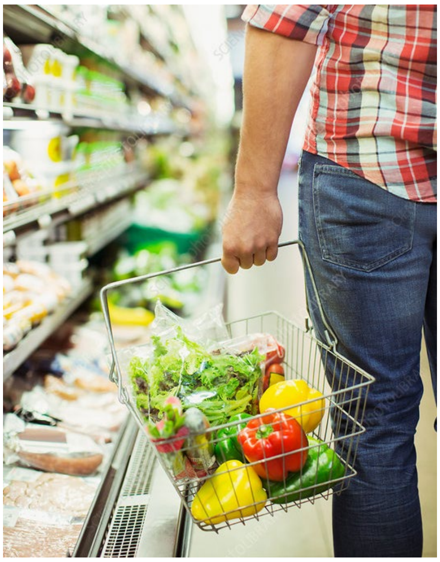

Image source

7.4.1.2 Task set-up & description

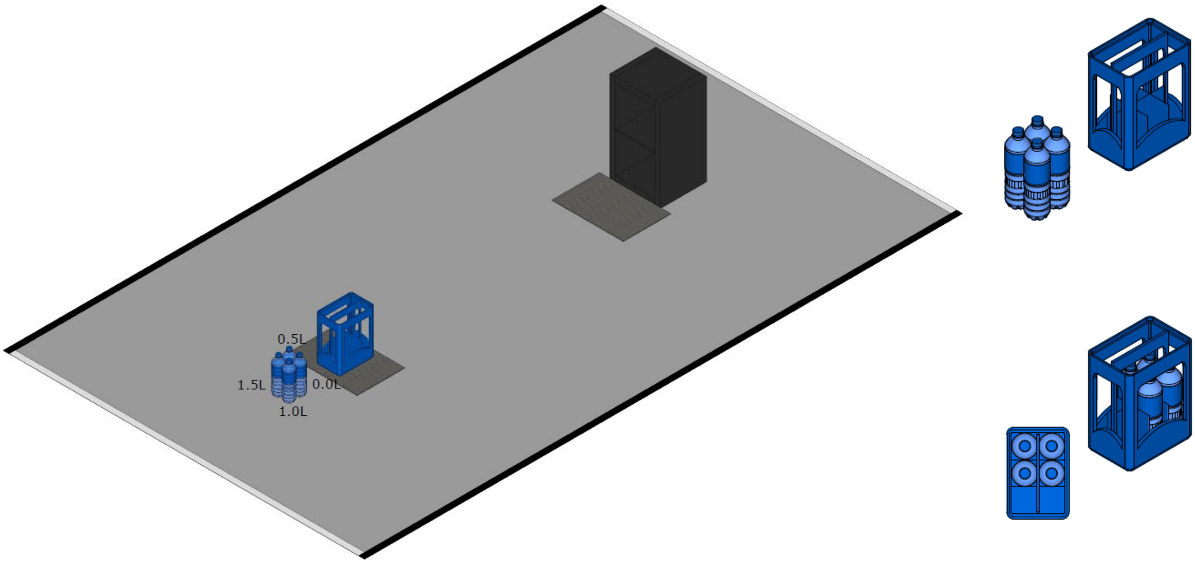

Pilots must carry the blue bottles using the blue bottle crate and then place the bottles on top of the shelf. The initial position of the 1.5 L PET bottles with the different fillings (1.5 L, 1.0 L, 0.5 L, 0 L water) is defined as in the illustration.

7.4.1.3 Task rules

ARM-BOT-1 The blue bottles must be carried to the shelf using the blue crate. If the blue bottles are carried without the crate, the task is failed.

Comment on ARM-BOT-1: The order of placing the bottles in the crate is free.

ARM-BOT-2 Each time the blue crate is put down on a mat, the crate must be placed on the mat in its entirety. If the crate is placed on the shelf or on the floor (next to the mats), the task is failed.

ARM-BOT-3 All blue bottles must be standing upright on the shelf and the blue crate must be standing on the mat closer to the finish line, when the pilot crosses the finish line of the task.

ARM-BOT-4 If any of the blue bottle touches the ground (floor or mat) again, after it has been lifted off the ground, the task is failed.

ARM-BOT-5 If any blue bottle tips over and touches the ground (floor or mat), the task is failed.

ARM-BOT-6 It is allowed that the crate slightly touches the side of the leg or hip while the pilots carry the crate with the prosthetic hand. If any part of the body (e.g., the legs or torso) is used to proactively stabilize the crate or the bottles when carrying the crate, the task is failed.

7.4.2 Serving Food

7.4.2.1 Introduction

Cooking often involves grasping and carrying objects (e.g., pans) of significant weight from one location to another while it must be made sure that none of the content is spilled.

In this task, a casserole dish and a frying pan must be carried from the stove to a predefined location on a table.

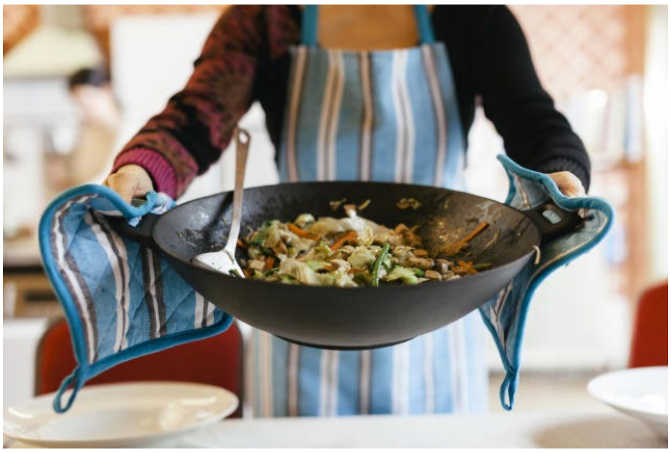

Image source

7.4.2.2 Task set-up & description

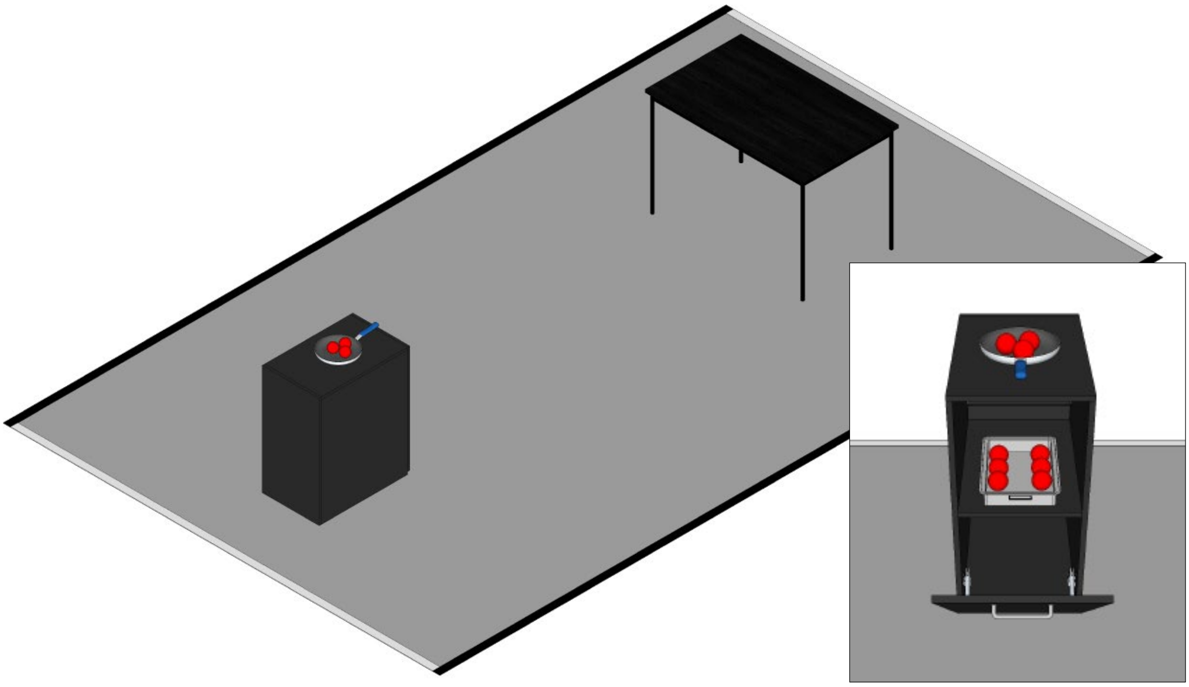

Pilots must carry a casserole dish and a frying pan from the oven to the table.

The frying pan and the casserole dish both contain weights in the form of lacrosse balls (three in the frying pan and six in the casserole dish). The balls can move around freely in their containers. The handles of the casserole dish are lifted all the way to the stop.
Insert: The casserole dish is initially placed in the oven. The oven is initially closed, the open oven is shown for illustration only. The handle of the oven is silver and can be opened with either hand.

7.4.2.3 Task rules

ARM-SERV-1 The frying pan and the casserole dish must be placed on top of the table.

ARM-SERV-2 The handle of the frying pan is blue. The rest of the frying pan is not allowed to be touched with the prosthetic or non-prosthetic hand.

ARM-SERV-3 The casserole dish must be held and carried at both handles when the casserole dish leaves the oven, i.e. the posterior handle crosses the front edge of the shelf.

ARM-SERV-4 If a lacrosse ball falls out of the frying pan or the casserole dish, the task is failed.

7.4.3 Storing Dishes

7.4.3.1 Introduction

Kitchen work oftentimes includes manipulating objects in confined space and at various heights, e.g., when grasping objects that are placed inside a cupboard.

In this task, typical kitchen objects must be grasped and stowed away at predefined target locations.

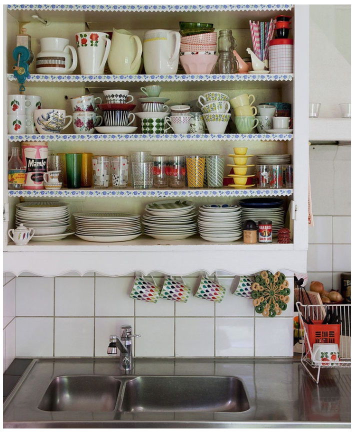

Image source

7.4.3.2 Task set-up & description

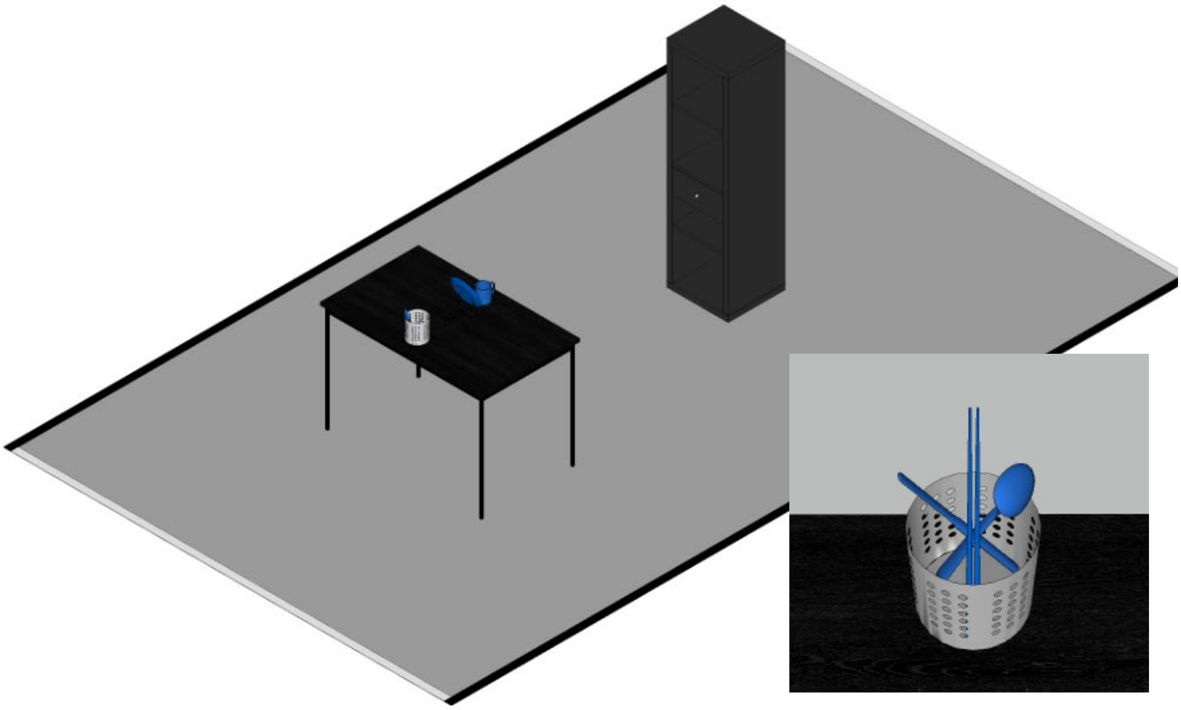

Pilots must move the blue dishes and blue cutlery from the table to their respective target locations in the shelf. Target locations are marked with dashed outlines. Close-up: Positioning of the cutlery in the tray in the drying stand.

Below left: frontal view of the shelf with all items at their target location

Below right: top view of the open drawer with all items at their target location.

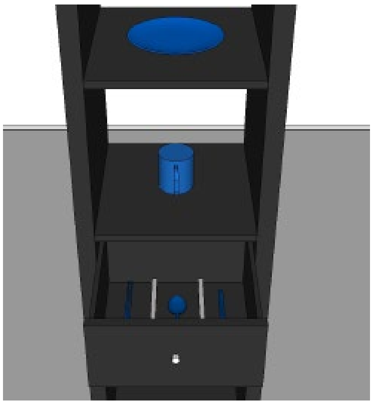

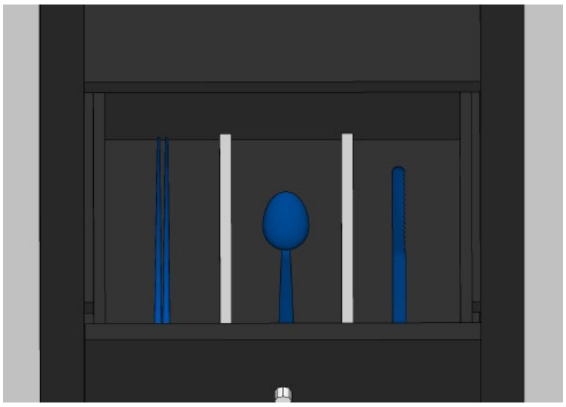

7.4.3.3 Task rules

ARM-DISH-1 The blue cutlery must be removed from the cutlery stand and stowed away in the corresponding cutlery tray compartment in the shelf.

ARM-DISH-2 The blue plate must be removed from the plate holder and placed on the top shelf.

ARM-DISH-3 The blue cup must be placed on the middle shelf.

ARM-DISH-4 The plate holder and the cutlery stand can be moved around on the table. If either of them is lifted off the table, the task is failed.

7.4.4 Hanging Laundry

7.4.4.1 Introduction

Handling laundry and putting on clothes requires a distinct set of fine motor skills, in particular with the fingers. Furthermore, for an arm prosthesis to be practical for daily use it must be compatible with standard clothes.

In this task, the pilot must put on a hooded sweater, fully close the zipper, take it off and hang it over the clothesline. Finally, the pilot must hang up a t-shirt on the clothesline using a blue clothespin.

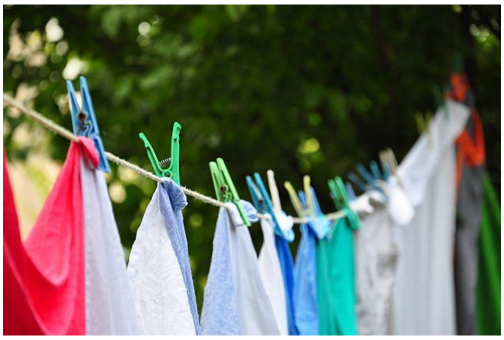

Image source

7.4.4.2 Task set-up & description

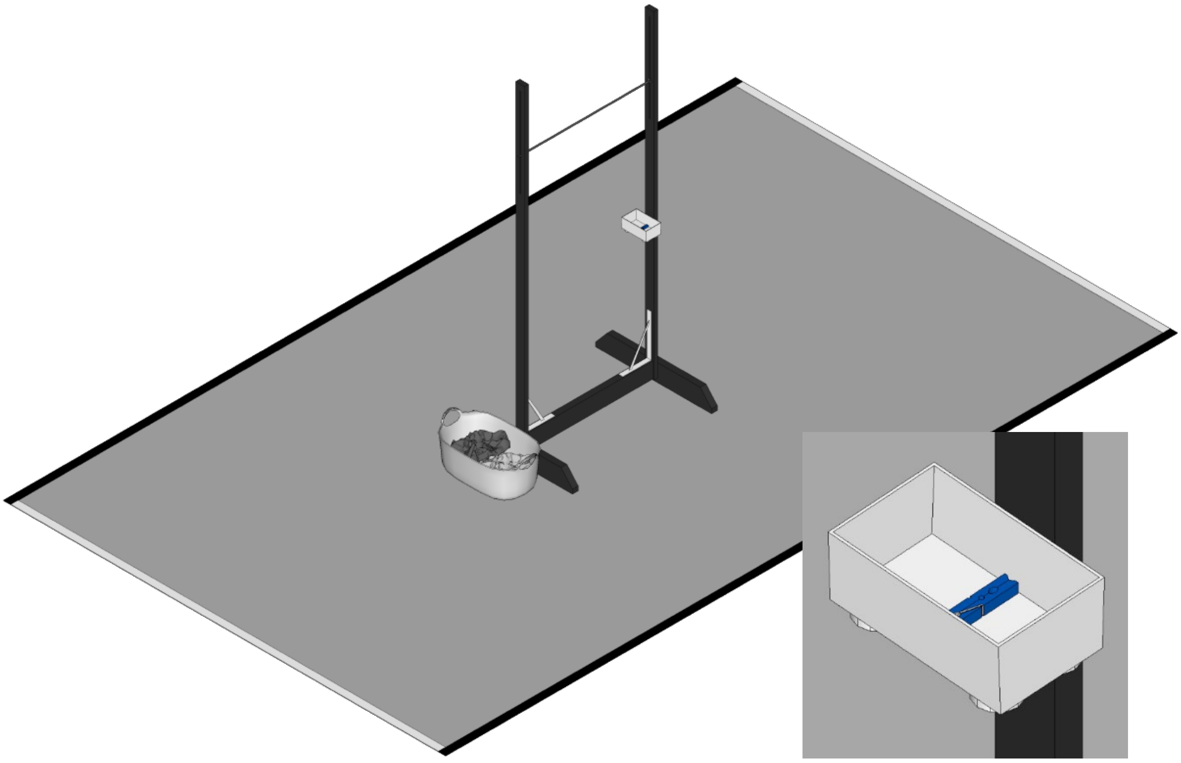

Pilots must hang a t-shirt on the clothesline using a blue clothespin, put on a hooded sweater, and hang it over the clothesline. The hooded sweater has a blue zip slider. Insert: Initial position of the clothespin in the box.

- Initially, the t-shirt and the sweater are randomly placed in the hamper.
- The sweater has a blue zip slider and is initially open. The sweater will be selected according to the size of the pilot (XS / S / M / L / XL / XXL).
- The height of the clothesline will be the pilot's body height + 0.1 m.

7.4.4.3 Task rules

ARM-LAUNDRY-1 The pilot must go through the following steps with the sweater. If any step is not executed correctly, the task is failed.

- The sweater must be put on correctly (both arms inserted fully through the sleeves).
- The zipper must be closed above the mark when wearing the sweater.
- The zipper must be opened completely to take off the sweater.
- The sweater must hang on the clothesline, when the pilot crosses the finish line of the task (e.g., throw the sweater over the clothesline).

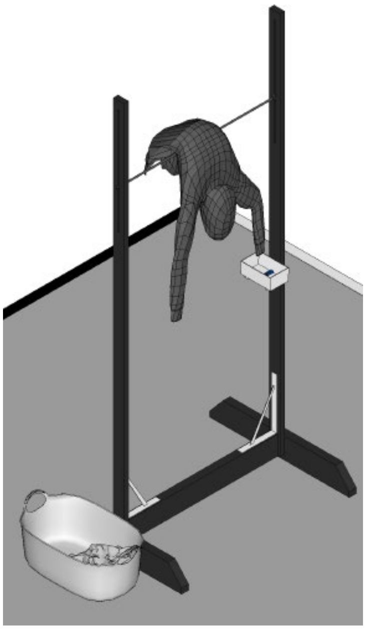

Comment on ARM-LAUNDRY-1: The referee will confirm if the zipper is closed above the mark with "Okay go", and the pilot may continue.

ARM-LAUNDRY-2 The blue zip slider is only allowed to be pulled by the prosthetic hand.

Comment on ARM-LAUNDRY-2: For inserting the zipper, the non-prosthetic hand can touch (but not grasp) the blue zip slider and the non-blue parts of the zipper.

ARM-LAUNDRY-3 The t-shirt must be hung on the clothesline. The blue clothespin must clamp the t-shirt and the clothesline. If the t-shirt is not attached with the blue clothespin to the clothesline when the pilot crosses the finish line, the task is failed.

Comment on ARM-LAUNDRY-3: It is permitted to hang the t-shirt over the line and then attach it with the blue clothespin.

7.4.5 Do-it-yourself

7.4.5.1 Introduction

The dexterous use of hand tools requires a prosthetic hand and wrist that provide active motion about multiple axes (pronation/supination, palmar flexion, dorsal extension, and radial and ulnar abduction). Since hand tools are often used in confined space the active control of many degrees of freedom becomes even more important.

In this task, pilots must use a variety of hand tools in the context of do-it-yourself type work.

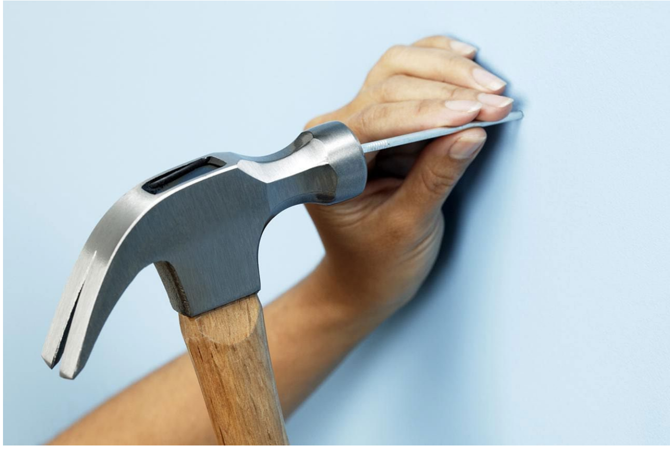

Image source

7.4.5.2 Task set-up & description

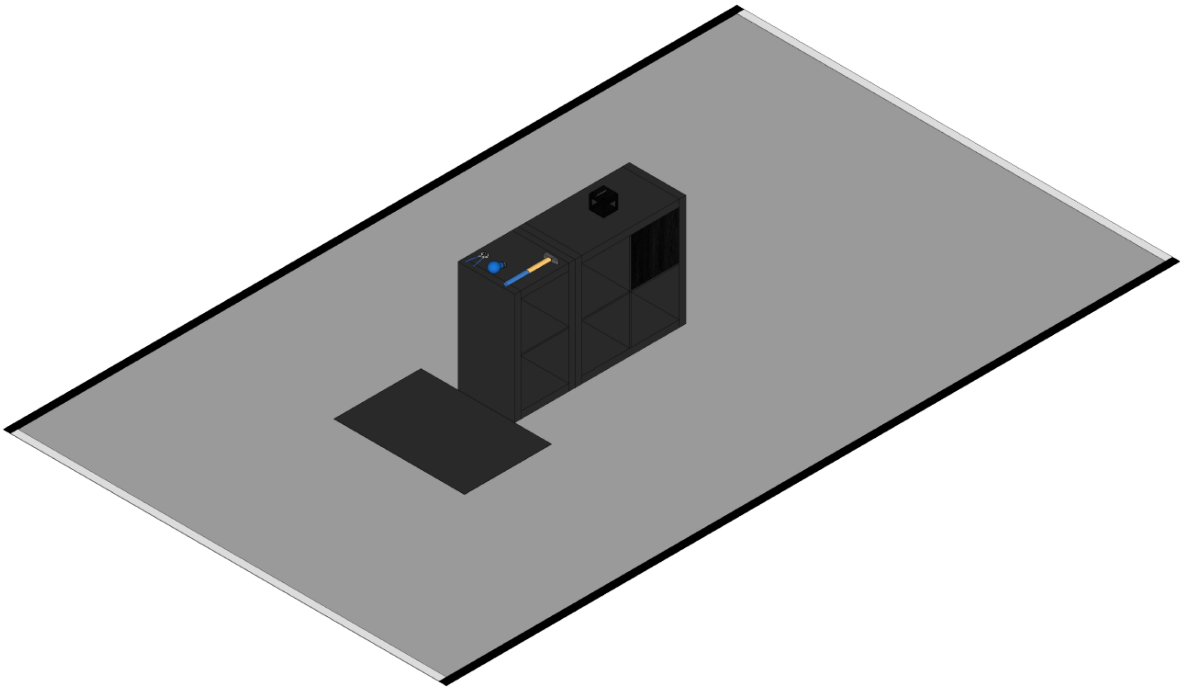

Pilots must drive a nail into a piece of 'wood' using a hammer and remove it using pliers. The handles of both the hammer and the pliers are blue. In addition, a blue light bulb must be screwed into a holder.

Below left: Initial location of the task objects. Below right: task objects after completion of all subtasks. The tools can be located anywhere on the top surface of the shelf.

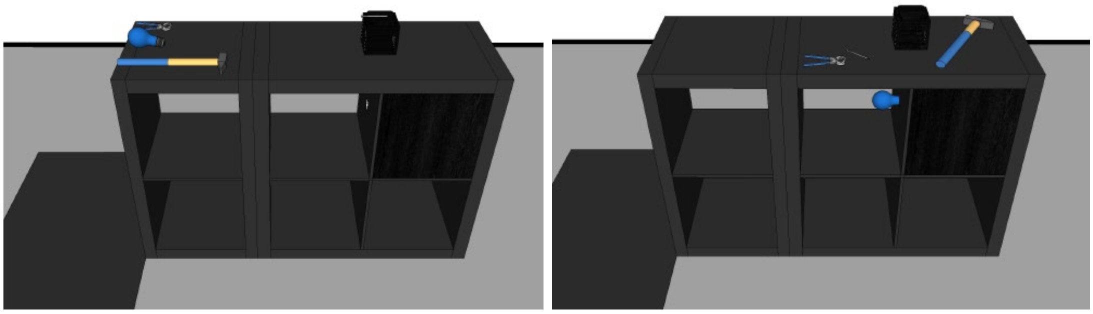

7.4.5.3 Task rules

ARM-DIY-1 The nail must be driven into the 'wood' (represented by a 3D printed plate) using only the blue hammer until its tip breaks the bottom surface of the plate. Once the nail has broken the bottom surface of the plate, the nail it must be fully removed from the wood using only the pliers.

Comment on ARM-DIY-1: The referee will confirm the breaking of the tip through the bottom surface of the plate with "Okay go".

ARM-DIY-2 The blue lightbulb must light up after it is screwed into the bulb holder.

Comment on ARM-DIY-2: In case of a technical defect, the referee can confirm the correct montage of the lightbulb with "Okay go".

ARM-DIY-3 It is not allowed to touch the non-blue parts of the tools or the light bulb with the prosthetic or non-prosthetic hand.

ARM-DIY-4 While standing on the mat with both feet in their entirety, it is allowed to use the non-prosthetic hand to support positioning the tools or the light bulb in the prosthetic hand. For the positioning while standing on the mat, the non-prosthetic hand can touch the blue parts and the non-blue parts of the tools or the light bulb.

Comment on ARM-DIY-4: It is not allowed to touch the tools or the light bulb with the non-prosthetic hand while standing off the mat.

ARM-DIY-5 If the 3-D printed plate is pulled off the fixation, the task is failed.

Comment on ARM-DIY-5: We recommend pushing down and stabilizing the 3D-printed plate with the non-prosthetic hand.

7.4.6 Containers

7.4.6.1 Introduction

The ability to use kitchen utensils (e.g., cutlery, a can opener) is critical for independent living and involves countless tasks which are typically solved by dexterous bimanual interaction. Also, some objects in the kitchen are very delicate to handle and require a very precise control of grip force.

In this task, pilots must conduct a series of kitchen related bimanual tasks.

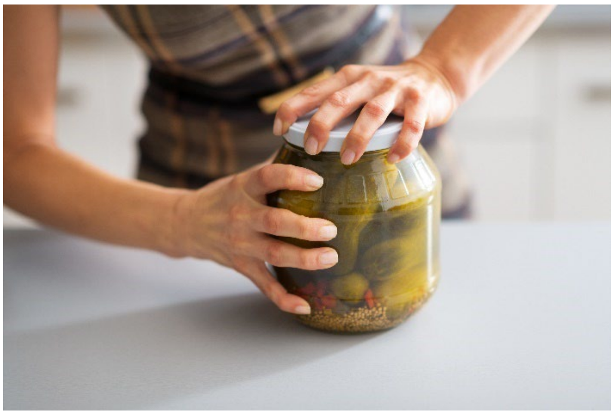

Image source

7.4.6.2 Task set-up & description

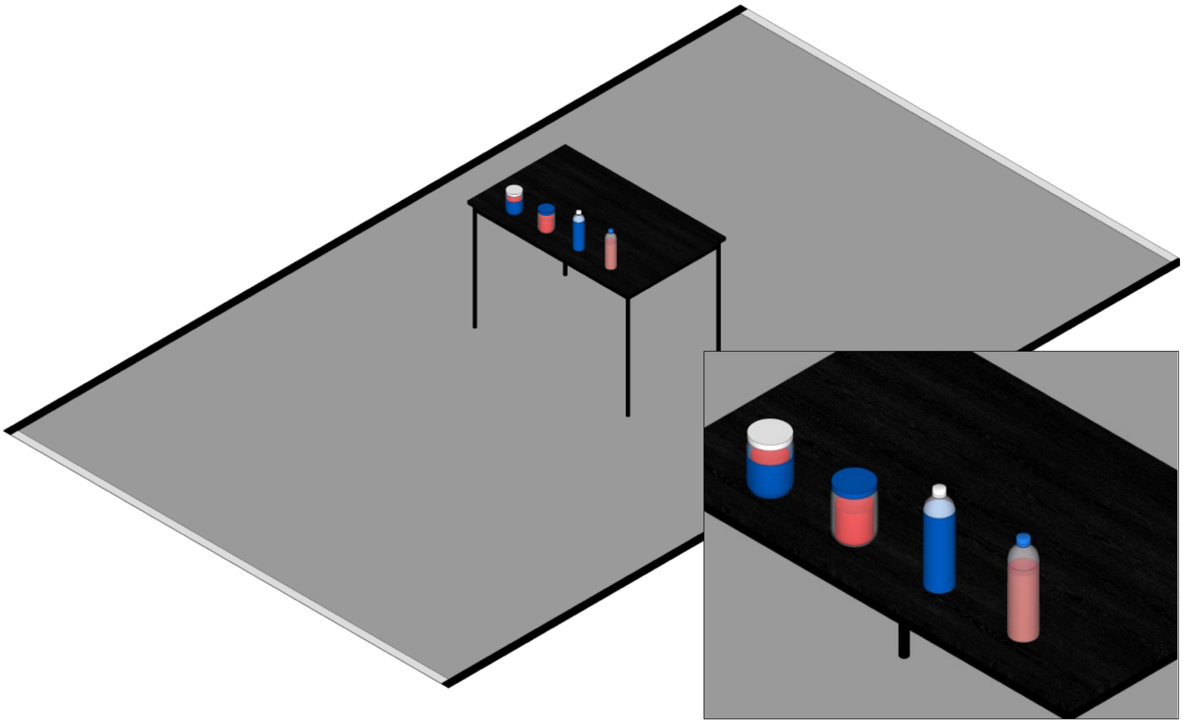

Pilots must open two bottles and two jam jars. Insert: Close-up of all containers on the table.

Description of the containers:

- One bottle has a blue lid, and the other has a blue bottle body.
- One jar has a blue lid, and the other jar has a blue jar-body.
- All containers are filled with red coloured water.

7.4.6.3 Task rules

ARM-CONT-1 The blue cap of the transparent PET bottle must be removed. If any of the content of the bottle is spilled the task is failed.

ARM-CONT-2 The white cap of the blue PET bottle must be removed. If any of the content of the bottle is spilled the task is failed.

ARM-CONT-3 The blue lid must be separated from the transparent jar. If any of the content of the jar is spilled, the task is failed.

ARM-CONT-4 The white lid must be separated from the blue jar. If any of the content of the jar is spilled, the task is failed.

7.4.7 Haptic Bag

7.4.7.1 Introduction

The availability of sensory feedback can improve a user's control over the prosthesis and increases the acceptance and embodiment of the device.

In this task, pilots must recognise and retrieve objects of different shapes and compliance in the absence of visual feedback.

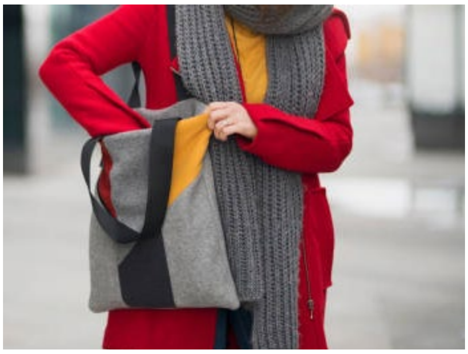

Image source

7.4.7.2 Task set-up & description

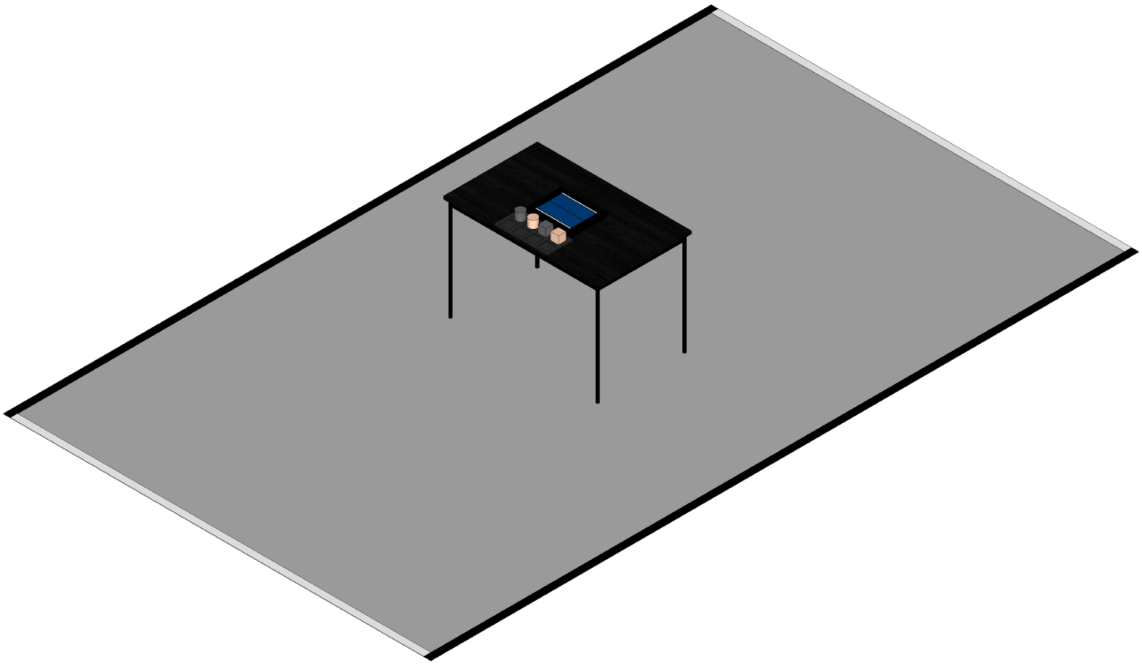

Pilots must reach into the bag only with their prosthetic hand to retrieve the objects in a predefined order (1-4, see below).

Four different objects are placed in the bag: a hard cylinder, a soft cylinder, a hard cube and soft cube (grey: hard, apricot soft). The order of the target objects on the rack is randomised. The pilots have no sight of their workspace during shape and compliance exploration and identification.

Below left: Example of randomized starting position. Below right: Corresponding target position.

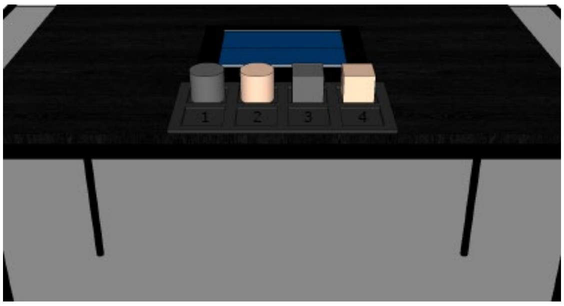

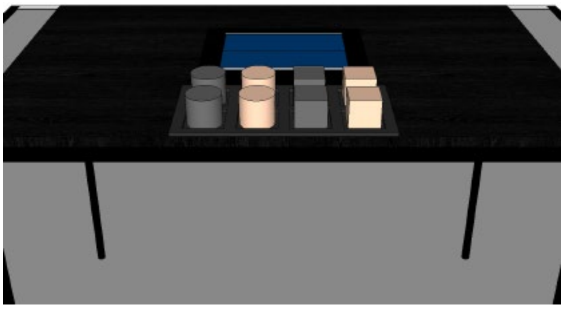

7.4.7.3 Task rules

ARM-HAPT-1 The objects must be removed from the bag in the order in which they are initially presented on the table (1-4).

ARM-HAPT-2 Only the prosthetic hand must be inserted into the bag through the blue opening. The bags must not be touched with the other hand.

ARM-HAPT-3 It is not allowed to push the objects inside the bag against the table or the edges of the haptic bag in order to deduce the compliance or shape of the objects.

ARM-HAPT-4 Only one object at a time must be removed from a bag.

ARM-HAPT-5 If a wrong object is removed at least partly from the bag (the referee sees the object), the task is failed.

ARM-HAPT-6 All forms of imaging and sensors are allowed to detect the objects (including sensors that use visible light, electromagnetic waves, lasers or similar).

ARM-HAPT-7 It is not allowed to intentionally look into the bag.

ARM-HAPT-8 The feedback to the pilot is not allowed to be the streamed image from inside the bag.

Comment on ARM-HAPT-8: The feedback to the pilot is allowed to be visual (e.g., icons or text representing the objects), auditive or haptic.

7.4.8 Hot Wire

7.4.8.1 Introduction

Maintenance of a tight grip during sustained postural changes of the arm (e.g., pronation and supination of the forearm, elbow flexion and extension) can be challenging for prosthetic hand users but is relevant in many situations in daily life (e.g., when picking up the phone or painting).

Pilots hold a conductive wire loop with a blue handle. A curved metal wire must be tracked without touching the wire with the loop by using the prosthetic hand only.

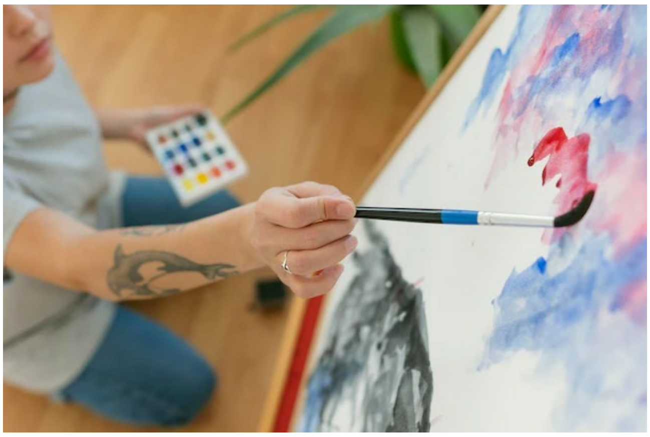

Image source

7.4.8.2 Task set-up & description

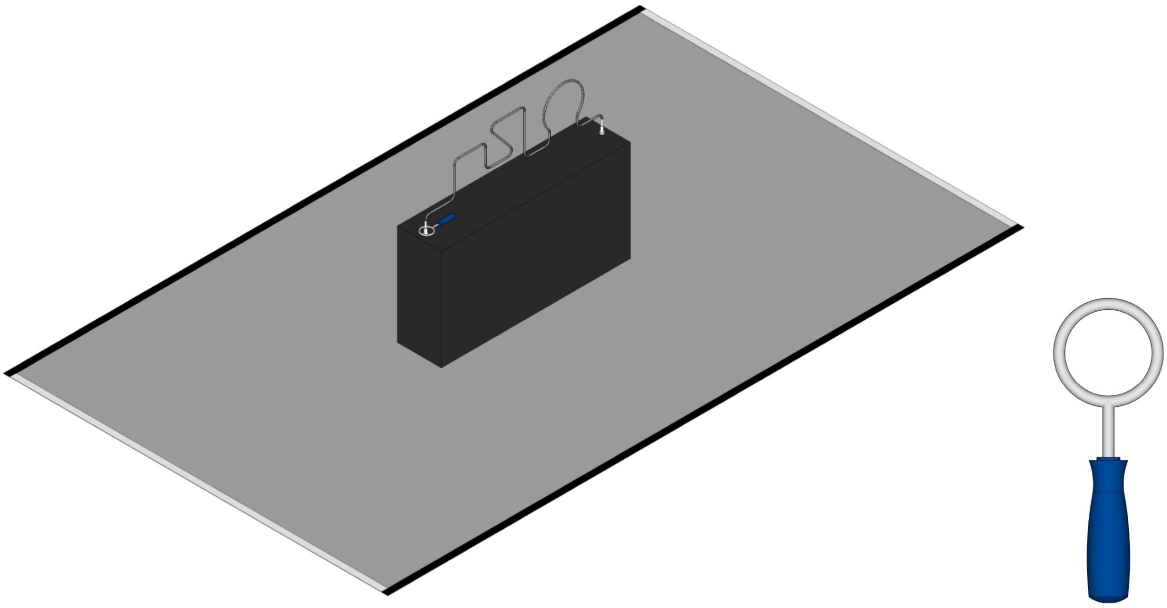

Pilots must move the metal loop with the blue handle around the wire to reach the target location, without touching the wire. The start and the target area of the bent wire are marked with white insulating plastic.
Insert: Close-up of the metal loop with the blue handle .

7.4.8.3 Task rules

ARM-WIRE-1 The task must be started and finished in the designated regions (start area and finish area), where pilots grasp and drop the loop respectively.

ARM-WIRE-2 If there is any contact between the loop and the wire when the loop is located outside the start or finish area, the handle lights up and the task is failed.

Comment on ARM-WIRE-2: In case of a technical defect, the referee can confirm the contact between the wire and the loop with “Task fail”.

ARM-WIRE-3 It is allowed to touch the silver part of the handle with the non-prosthetic hand to support positioning the handle while the loop is in the start area of the bent wire.

ARM-WIRE-4 If the silver part of the handle or the loop is touched with the prosthetic or non-prosthetic hand outside of the start and finish area, the task is failed.

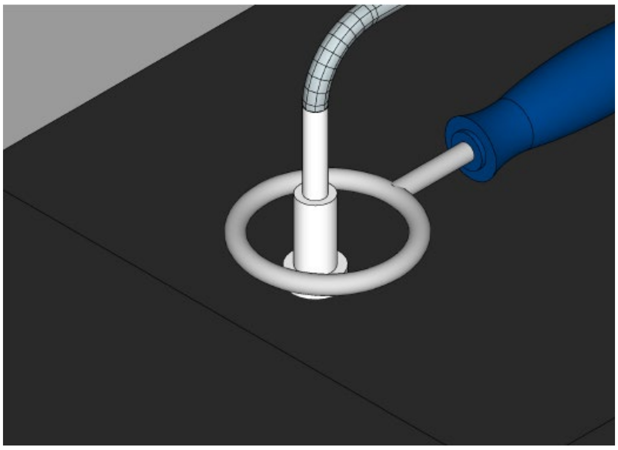

Start area (white part)

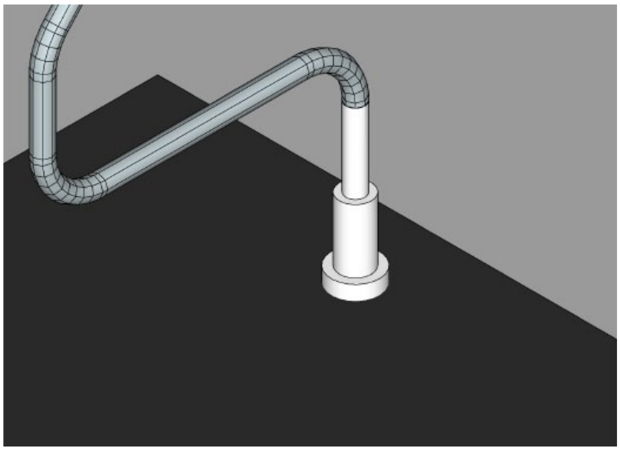

Finish area (white part)

7.4.9 Stacking

7.4.9.1 Introduction

Maintenance of a tight grip during postural changes of the arm (e.g., pronation and supination of the forearm, elbow flexion and extension) can be challenging for prosthetic hand users but is relevant in many situations in daily life such as when pouring liquids or turning objects.

In this task pilots sit in front of a table and must stack blue cups to a vertical pyramid.

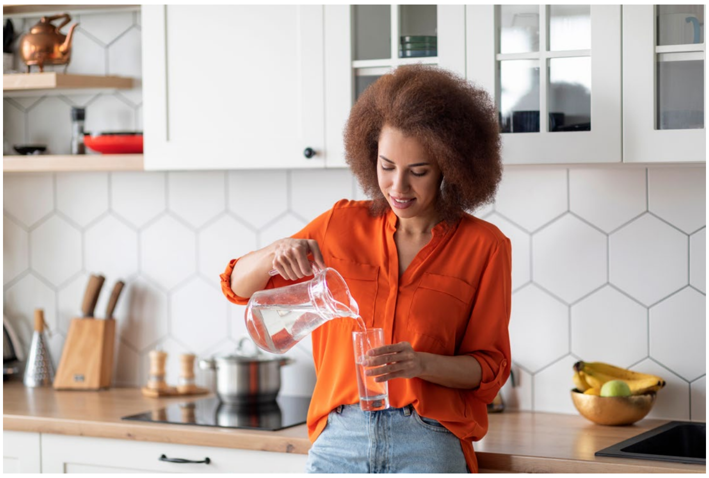

Image source

7.4.9.2 Task set-up & description

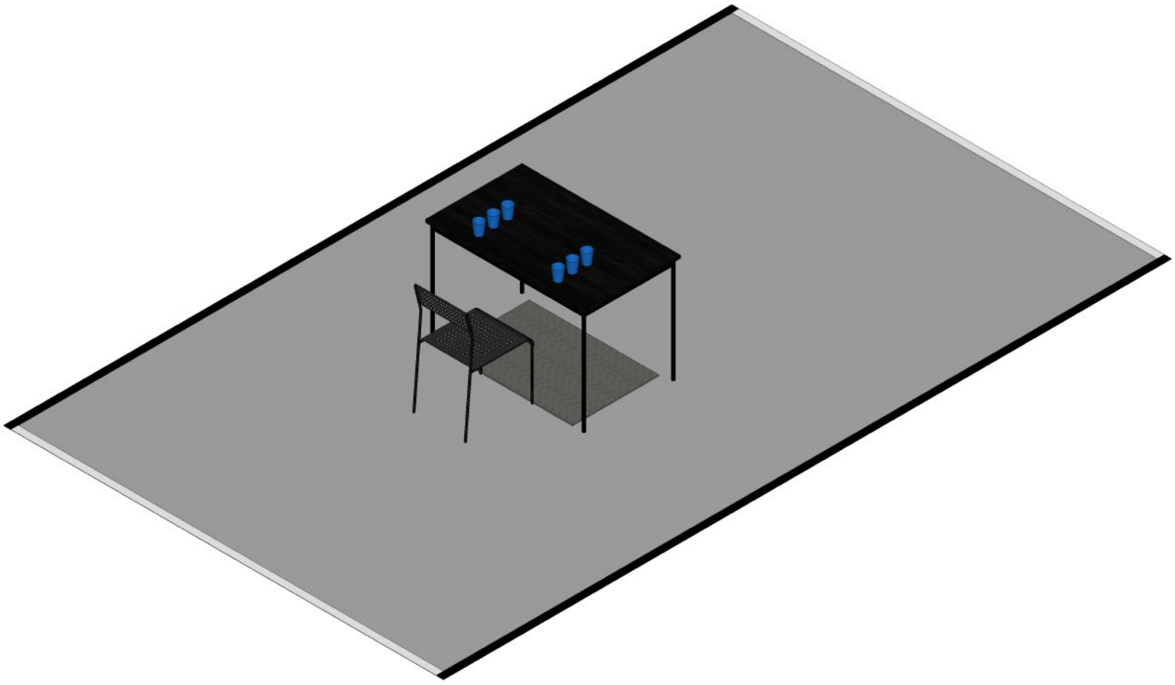

Pilots must stack the cups to a pyramid and then dissemble them again.

Below left: initial position of the blue cups; centre: blue cups stacked to a vertical pyramid; right: blue cups stacked to a single pile.

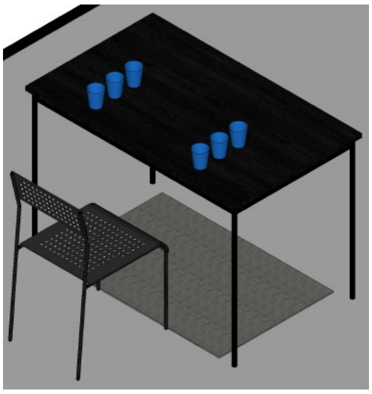
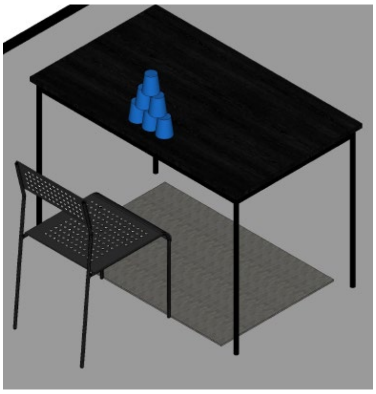
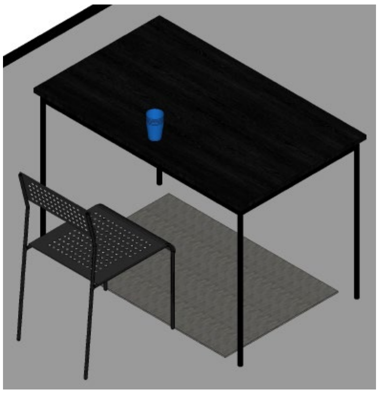

7.4.9.3 Task rules

ARM-STACK-1 All blue cups must be stacked to a three-level vertical pyramid (3-2-1) on the table. The opening of the blue cups must face downwards in the pyramid.

ARM-STACK-2 The pilot must be sitting on the chair while stacking and disassembling the blue cups. To ensure sitting, from the first touch of a cup, the pilot's feet (in their entirety) must be placed on the mat below the table.

Comment on ARM-STACK-2: The pilot is allowed to move the chair to comfortably sit down.

ARM-STACK-3 After stacking the blue cups to a pyramid, the pilot must place both hands simultaneously on the table. Thereafter the pyramid must be disassembled, and the blue cups must be stacked to a single pile of cups.

Comment on ARM-STACK-3: The referee confirms the placement of the two hands on the table with "Okay go".

ARM-STACK-4 The single pile of blue cups must be standing on the table with the opening facing upwards when the pilot crosses the finish line of the task.

ARM-STACK-5 If the lateral surface of any blue cup touches the table (e.g., after it drops), the task is failed.

Comment on ARM-STACK-5: It is not considered a task fail if a blue cup drops on the table and by chance stops still on its opening or bottom without tipping over.

7.4.10 Clean Sweep

7.4.10.1 Introduction

A vast variety of objects of different shape, size, compliance, texture, and weight must be grasped and manipulated in everyday life. The ability to cope with this diversity of requirements is challenged in this task.

In this task, pilots are asked to grasp and move blue objects individually with their prosthetic hand from their random, initial position on a table surface to a target position on a neighbouring table.

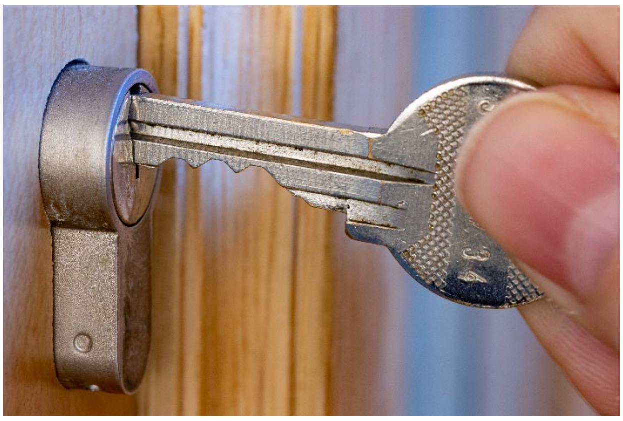

Image source

7.4.10.2 Task set-up & description

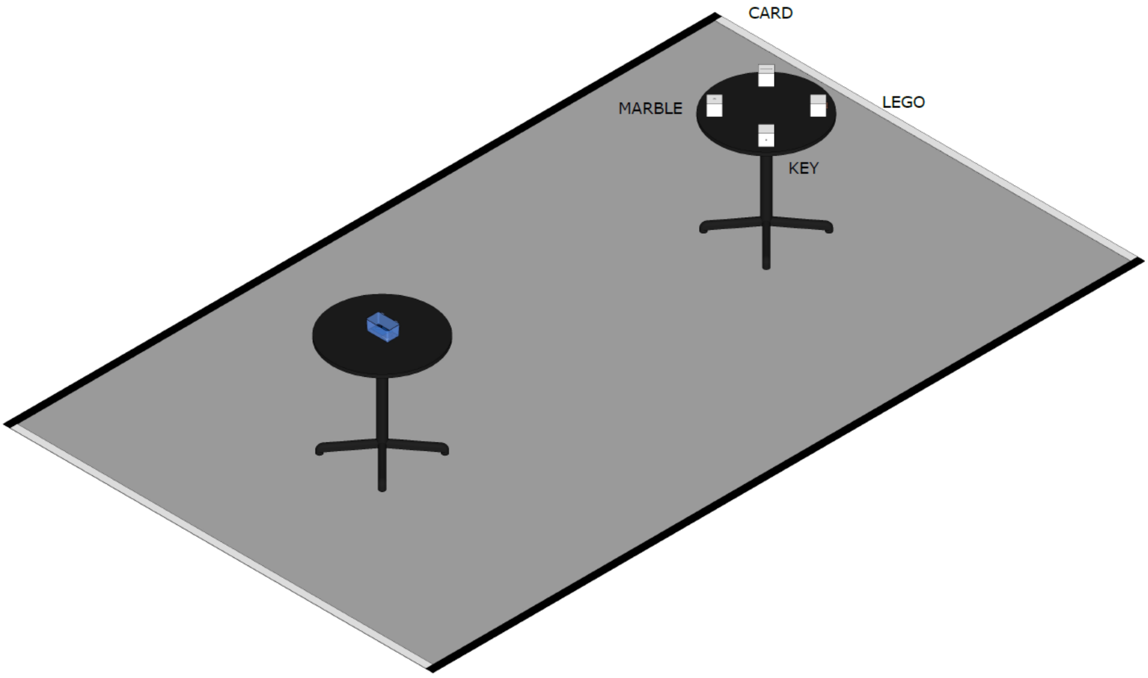

The blue objects located on the table near the start line must be placed at their respective target positions on the table near the finish line.

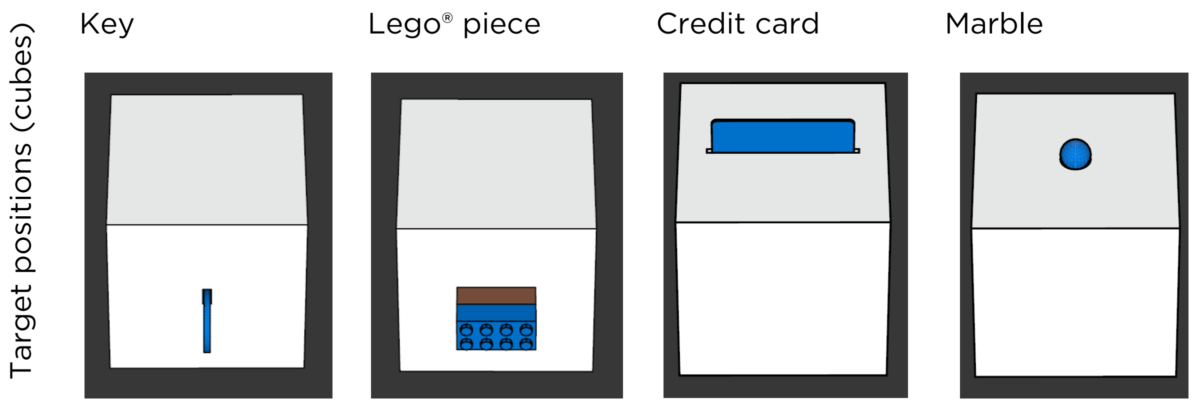

7.4.10.3Task rules

ARM-CLEAN-1 All blue objects must be moved from their random initial position in the blue box on the table near the start line to their designated target position on the table near the finish line.

Comment 1 on ARM-CLEAN-1: The target positions (cubes) are fixed on the table (e.g., with tape or screws). It is allowed to stabilize the target position on the table using the non-prosthetic hand, but it is not allowed to intentionally move or lift the target position off the table surface.

Comment 2 on ARM-CLEAN-1: The long side of the box is parallel to the start line. There is no lid on the box.

ARM-CLEAN-2 It is not allowed to transport the blue objects using the blue box. While the pilot is placing blue objects to their designated target position, the blue box must remain on the table near the start line.

ARM-CLEAN-3 If not all blue objects are located at their designated target position on the table near the finish line when the pilot passed the finish line of the task, the task is failed.
