# Supplementary material for: HANDSON Hand: Strategies and Approaches for Competitive Success at CYBATHLON 2024
Source: Bioengineering (Basel). 2025 Feb 24;12(3):228. doi: 10.3390/bioengineering12030228 (PMC11939478; doi:10.3390/bioengineering12030228)
Supplement: Supplementary file 1 [file bioengineering-12-00228-s001.zip › S3. Device_Description.pdf]

CYBATHLON
Risk Management File

Date 14 March 2022
VersionV_2022_03_14
Author(s) Lukas Jaeger

Table of Contents

1. Team and Event Information 3

2. Device identification 4
   2.1. Description 4
   2.2. Assembly definition of the element 5
   2.3. Assembly identification 5
   2.4. Specification 6
   2.5. Involved people 6

A. General safety measures 8

B. Specific risks and failures, detection and preventing measures 10
   2.6. Critical Risk Priority Number 11
   2.7. Factors of the Risk Priority Number (RPN) 11
      2.7.1. S – Severity 12
      2.7.2. O – Occurrence 12
      2.7.3. D – Detection 13

3. Detailed Risk Analysis 1
   3.1. Assembly 1 14

1. Team and Event Information

Teams are required to submit full documentation of their device for each CYBATHLON event participation.

Please insert the following information and sign at the bottom of the page.

**Team name**	HANDSON
Device name	HANDSON HAND
**Device brand (if applicable)**	--
**Team corresponding person name**	Dr. Xuhui Hu
**Team corresponding person e-mail**	brain94hxh@gmail.com

Please indicate below the event for which you submit the documentation:

**Type of event**	**CYBATHLON Experience**		**CYBATHLON Series**		**CYBATHLON Challenges**		**CYBATHLON 2024**	X
**Location of event**	Zurich							
**Date of event**	25-27 October 2024							

Responsibility and Approval

I hereby confirm that the risks and associated mitigation measures indicated in this Risk Management File have been assessed and implemented to the best of our knowledge to ensure safe operation of the device at the CYBATHLON races or on the CYBATHLON racetracks:

**Responsible person**	Dr. Xuhui Hu
**Role of responsible person within the project**	Lab head
**Place and date**	Nanjing, 17 August 2024
**Signature (wet-ink)**	....

2. Device identification

2.1. Description

Describe your device here. i.e. What is its intended purpose?, Who is the intended user?, How does it work?, What can it do?, What does it look like (picture, drawing, etc.)?

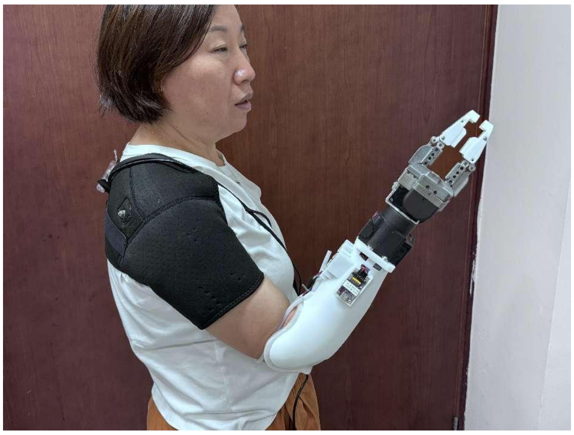

Wearing(Hand)

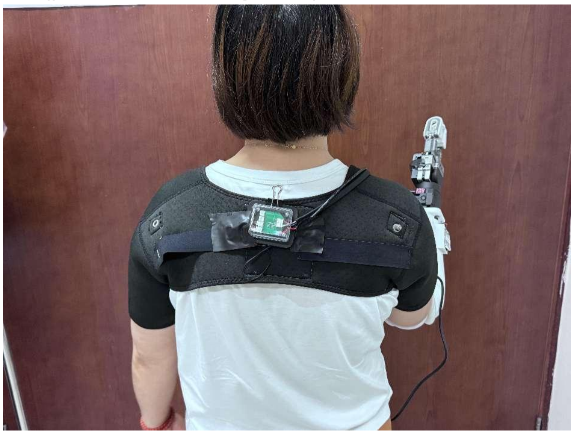

Wearing(Body-driven Controller)

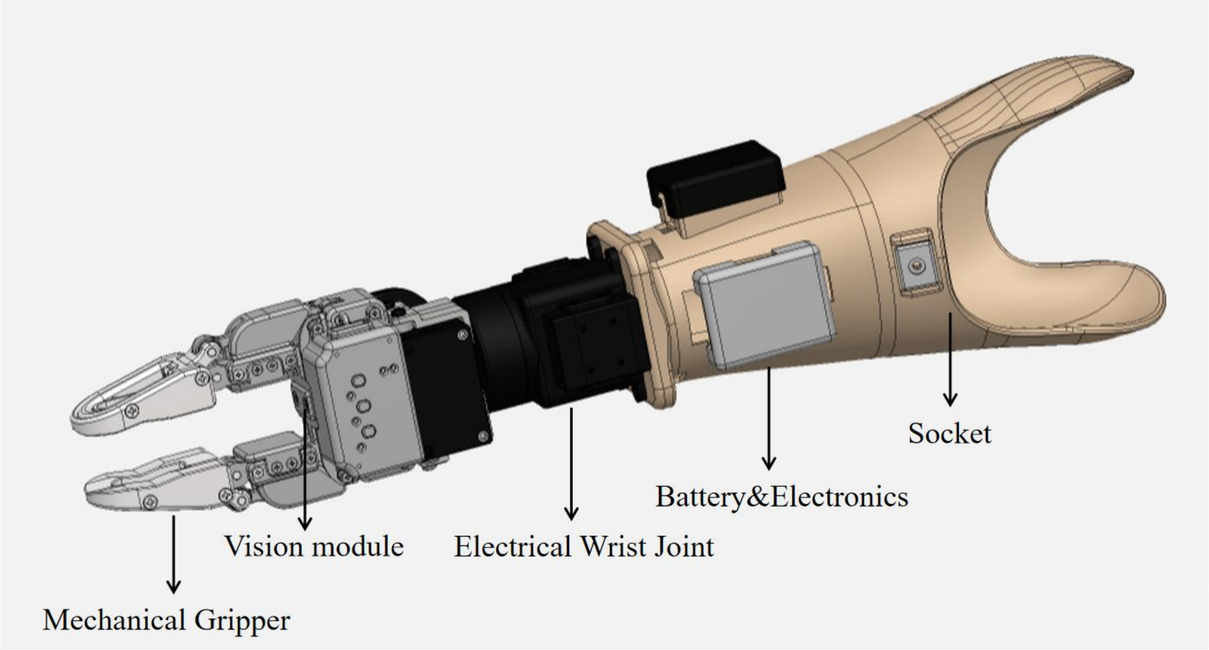

Sectional View(Hand)

The HANDSON Hand is a hybrid-driven prosthetic hand prototype. The hand is designed to better release the disabled person's ability to control multiple arm joints and to enhance wearing comfort and control reliability for everyday tasks such as grasping or manipulating objects. The prosthetic hand joint has a body-driven active degree of freedom to enable the opening and closing of the hand by conventional body-driven control method. At the same time, the motor-driven wrist is controlled by EMGs. In order to accomplish the haptic bag task, a vision module is installed on the mechanical gripper to detect different objects. The pilot use prosthetic limbs to pick up target objects without visual contact according to the programmed sound and light feedback.

2.2. *Assembly definition of the element*

Describe individual assemblies and components here, suggested format as follows:

Element

- Assembly 1
  - Part 1
  - Part 2
  - Part 3
- Assembly 2
  - Part 4
  - Part 5
  - Part 6
- Assembly 3
  - Part 7
  - Part 8

HANDSON Hand

- Hand
  - Electric palm
  - Electric wrist joint
  - Battery
  - Socket
  - Electrode
  - Circuit board
- Body-driven Controller
  - Belt
  - Tension sensor
  - Circuit board
  - Fixed bolt
  - Wire
- Vision Module
  - Camera
  - Neural network
  - Control Unit

2.3. *Assembly identification*

Identify assemblies and parts by images, sketches, etc.

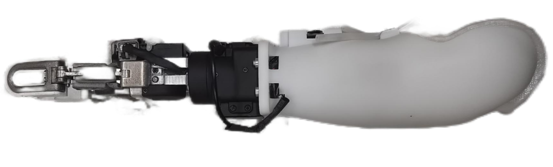

Hand(left view)

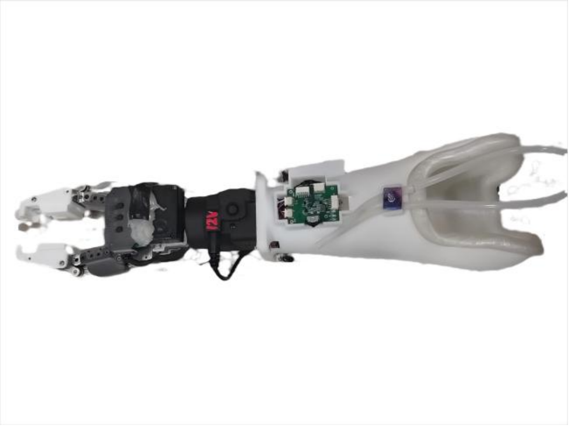

Hand(top view)

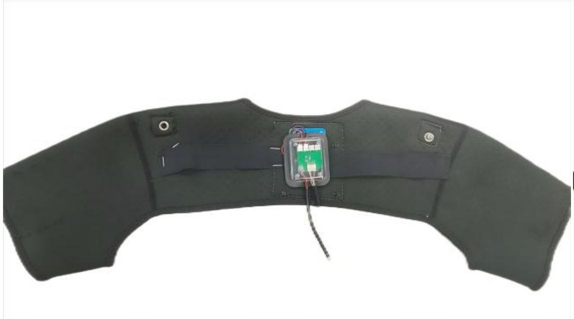

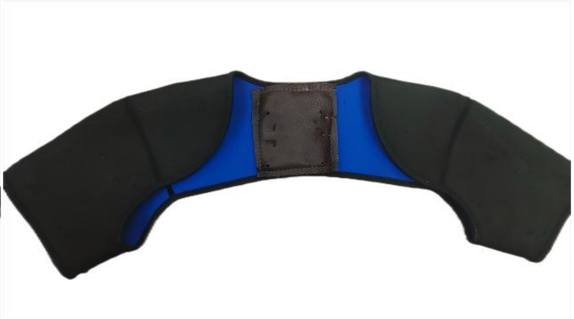

Body-driven Controller(front view)   Body-driven Controller(back view)

2.4. *Specification*

Specify each element in terms of size, weight, material, power requirement, power in-/output…

HANDSON Hand

Size: 150mm × 75mm × 50mm (Hand)
Weight: 350g (Hand)
Grasp Force: 170N
Operating Voltage: 12V
Maximum Power: 10W
Material Quality: Aluminium alloy, Stainless steel, Nylon

Body-driven controller

Size: 440mm × 100mm × 40mm
Weight: 50g
Operating Voltage: 5V
Maximum Power: 1W
Material Quality: Cotton, Plastic, Leather

Vision module

Size: 26mm × 12.5mm × 7mm
Resolution: 320*240
Frame rate: 30fps
Operating Voltage: 5V
Operating Current: 80mA - 120mA
Operating Temperature: 0°C to 50°C

2.5. *Involved people*

List the main people involved in the development of this device and their tasks in the project:

- Xuhui Hu (System Design)
- Fengkai Guo (File Compiling)

- Junfa Dai (Belt Design)
- Anran Li (Hand Design)
- Zhiyi Chen (Hand Design)
- Zhikai Wei (Vision Module Design)
- Min Xu (Pilot)

List the people involved in compiling the risk management file and their tasks in the project (ideally, the risk analysis is conducted by a minimum of two engineers and one person with a clinical background, e.g., physician or therapist):

- Xuhui Hu (Device identification)
- Fengkai Guo (Safety evaluation)
- Junfa Dai (Physician, Review the document)

A. General safety measures

Describe general precautions made to ensure safety of your device. Consider hardware, e.g., electronics or mechanical components, and software.

In terms of **general safety guidelines** for medical electrical equipment, you may check the following standards:

- IEC 60601-1:2005+AMD1:2012, *Medical electrical equipment - Part 1-1: General requirements for safety - Collateral standard: Safety requirements for medical electrical systems*

- IEC 60601-1-2: Ed. 4.0 b:2014, *Medical electrical equipment - Part 1-2: General requirements for basic safety and essential performance - Collateral Standard: Electromagnetic disturbances - Requirements and tests*

- IEC 60601-1-6: Ed. 3.1 b:2013, *Medical electrical equipment - Part 1-6: General requirements for basic safety and essential performance - Collateral standard: Usability*

- IEC 62366-1:2015, *Medical devices -- Part 1: Application of usability engineering to medical devices*

- IEC 60601-1-8: Ed. 2.0 b:2006, *Medical electrical equipment - Part 1-8: General requirements for basic safety and essential performance - Collateral Standard: General requirements, tests and guidance for alarm systems in medical electrical equipment and medical electrical systems.*

In terms of safety of **electroencephalographs**, you may specifically check

- IEC 60601-2-26: Ed. 3.0 b:2012, *Medical electrical equipment - Part 2-26: Particular requirements for the basic safety and essential performance of electroencephalographs*.

In terms of safety of **FES stimulators** and **bikes**, you may specifically check

- IEC 60601-2-10: Ed. 2.1 b:2016, Medical electrical equipment - Part 2-10: Particular requirements for the basic safety and essential performance of nerve and muscle stimulators

- ASTM F2711-08(2012), *Standard Test Methods for Bicycle Frames*.

In terms of safety of **prostheses and orthoses**, you may specifically check the ISO standards catalogue ISO/TC168, *Prosthetics and Orthotics*, in particular:

- ISO 10328:2006, *Prosthetics - Structural testing of lower-limb prostheses - Requirements and test methods.*

- ISO 22523:2006*, External limb prostheses and external orthoses -- Requirements and test methods*

Please note that this listing is not intended to be exhaustive.

The device is an arm prosthesis prototype. It is for testing/evaluating purposes only. The pilot is asked to follow the usage instructions when operating the device.
The motion control circuit board of the prosthetic hand is shielded properly. Unsafe movements due to external EMI is prevented.

Temperature protection is implemented into the device. The temperature of the touchable area of the device will not exceed 40℃. The user is sufficiently protected from extreme temperatures.

A start-up self-check function is implemented into the device control program. The device does start-up in safe mode. When no user input is given, the prosthetic hand will not perform any movements and the force is limited.
The maximum voltage of the battery pack used in the device is 8.4V. The maximum voltage of the device does not exceed 12V.

B. Specific risks and failures, detection and preventing measures

Each risk/failure should be listed in the "Detailed Risk Analysis" below. Describe the failure and possible resulting effects, rate the probability of its occurrence, the severity, and the probability to detect the failure. Describe preventing measures and rate the failure again.

The format of this risk/failure table has been adapted from:

http://www.npd-solutions.com/fmea.html,
http://www.harpcosystems.com/articles/Design-FMEA-Ratings-Part1/,
http://www.harpcosystems.com/articles/Design-FMEA-Ratings-Part2/,
http://www.harpcosystems.com/articles/Design-FMEA-Ratings-Part3/

Examples of possible hazards are listed below (based on ISO 14971):

**Examples of energy hazards**	**Examples of biological and chemical hazards**	**Examples of operational hazards**	**Examples of information hazards**
Electromagnetic energy<br>Line voltage<br>Leakage current<br>enclosure leakage current<br>earth leakage current<br>patient leakage current<br>Electric fields<br>Magnetic fields<br>Radiation energy<br>Ionizing radiation<br>Non-ionizing radiation<br>Thermal energy<br>High temperature<br>Low temperature<br>Mechanical energy<br>Gravity<br>falling<br>suspended masses<br>Vibration<br>Stored energy<br>Moving parts<br>Torsion, shear and tensile<br>Force<br>Moving and positioning of pilot<br>Acoustic energy<br>ultrasonic energy<br>infrasound energy<br>sound	Biological<br>Bacteria<br>Viruses<br>Other agents (e.g., prions)<br>Re- or cross-infection<br>Chemical<br>Exposure of airway, tissues, environment or property, e.g., to foreign materials:<br>acids or alkalis<br>residues<br>contaminates<br>additives or processing aids<br>cleaning, disinfecting or testing agents<br>degradation products<br>medical gasses<br>anaesthetic products<br><br>Biocompatibility<br>Toxicity of chemical constituents, e.g.:<br>allergenicity/irritancy<br>pyrogenicity	Function<br>Incorrect or inappropriate output or functionality<br>Incorrect measurement<br>Erroneous data transfer<br>Loss or deterioration of function<br>Use error<br>Attentional failure<br>Memory failure<br>Rule-based failure<br>Knowledge-based failure<br>Routine violation	Labelling<br>Incomplete instructions for use<br>Inadequate description of performance characteristics<br>Inadequate specification of intended use<br>Inadequate disclosure of limitations<br>Operating instructions<br>Inadequate specification of accessories to be used with the device<br>Inadequate specification of pre-use checks<br>Over-complicated operating<br>Instructions<br>Warnings<br>of side effects<br>of hazards likely with re-use of single-use medical devices<br>Specification of service and maintenance

2.6. *Critical Risk Priority Number*

During the risk analysis, each risk or failure is analysed and rated with respect to its severity (S), probability of occurrence (O), and detection rate (D). The rating for each of the three aspects ranges from 1 (low security risk/failure, low probability of occurrence, high detection probability) to 10 (severe injuries or death, high probability of occurrence, no/low probability for detection). The product out of these three ratings is called Risk Priority Number (RPN). In case, the RPN is greater than a critical threshold, preventing measures are required to reach a final RPN below or equal to the critical threshold by means of reasonable and justifiable security measures.

Define a critical threshold in this section here – we recommend a critical **RPN threshold of 75**.

In case, the risk is greater than the critical threshold the risk **must clearly be mentioned** in the "declaration of agreement" signed by the pilot and involved staff.

Compared with other electrical prosthetic hand and out of the experience with previous models a **Risk Priority Number, RPN = 75** seems to be a good compromise between safety, benefit and expense.

2.7. *Factors of the Risk Priority Number (RPN)*

Find below a recommendation how to rate occurrence, severity, and detection. The "Risk Priority Number before" is a mathematical product of the numerical Severity- (S), Occurrence- (O), and Detection-Ratings (D) obtained before applying any preventing measures to reduce the likelihood for dangerous incidents, thus: **RPN before = (S1) x (O1) x (D1)**. This "RPN before" should be set to prioritize items that require additional quality planning or action.

The "RPN after" is a mathematical product of the numerical Severity- (S), Occurrence- (O), and Detection-Ratings (D) obtained after applying the preventing measures to reduce the likelihood for dangerous incidents, i.e. **RPN after = (S2) x (O2) x (D2)**. The "RPN after" has to be equal or below the predefined threshold in order to guarantee safe use of the part/element/device.

Preventing measures are mechanisms that prevent the cause of the failure mode from occurring or that detect the failure and stop the application before an incident can happen. It could also reduce the severity by e.g. designing softer and rounder edges. Preventing measures could include specific inspection, testing or quality assurance procedures; selection of other components or materials; de-rating; limiting environmental stresses or operating ranges; redesign of the item to avoid the failure mode; monitoring mechanisms; performing preventative maintenance; or inclusion of back-up systems or redundancy.

2.7.1. S – Severity

Rating S	Criteria: Severity of effect	Consequence	Treatment
10	Death	-	-
9	Quadriplegia	Life-long medical care necessary / coma / permanent damage	Hospital stay
8	Amputations, paraplegia, blindness, deafness, traumatic brain injury (severe), fourth-degree burns	Life-long medical care necessary / coma / permanent damage	Hospital stay
7	Complex fractures, open fracture, inner injuries, traumatic brain injury (severe), third-degree burns	Permanent damage possible	Hospital stay
6	Gash, fractures, torn muscles, articular cartilage injury, traumatic brain injury (moderate), second-degree burns	Permanent damage possible	Hospital stay
5	Gash, fractures, torn muscles, articular cartilage injury, traumatic brain injury (mild), second-degree burns	Reversible injury	Hospital stay or ambulant treatment
4	Severe cuts, severe scratches, severe contusions, strains, first-degree burns	Reversible injury	Ambulant treatment or self-treatment
3	Minor cuts, minor scratches, minor contusions, stiff muscles, tension, blisters, excoriations, sickness, first-degree burns	Discomfort during application up to three days after application	Self-treatment
2	Slight sickness, pressure marks	Discomfort	-
1	No harm	-	-

2.7.2. O – Occurrence

Rating O	Criteria: Probability of occurrence
10	Occurs or may occur very likely during every use of the session
9	Occurs or may occur likely during every use of the session
8	Occurs in 1 of 5 sessions (less than once a day)
7	Occurs in 1 of 10 sessions (less than once a day)
6	Occurs in 1 of 50 sessions (less than once half a month)
5	Occurs in 1 of 100 sessions (less than once a month)
4	Occurs in 1 of 500 sessions (less than once half a year)
3	Occurs in 1 of 1000 sessions (less than once per year)
2	Occurrence very unlikely
1	Occurrence nearly impossible

2.7.3. D – Detection

Rating D	Criteria: Likelihood of detection by design control
10	No chance of detection
9	Very remote chance of detection
8	Remote chance of detection
7	Very low chance of detection by indirect methods (hardware or software)
6	Low chance of detection by indirect methods (hardware or software)
5	Moderate chance of detection by indirect methods (hardware or software)
4	High chance of detection by indirect methods (hardware or software)
3	High chance of detection by direct or indirect methods (hardware/software)
2	Direct and indirect detection: Hardware or software
1	Direct detection: Hardware or safe software (category 4, performance level e)

3. Detailed Risk Analysis

3.1. Prosthetic Hand

Assembly	Failure & Effect	S1	O1	D1	RPN before	Preventing measures	S2	O2	D2	RPN after
Servo	Overheating of the motor due to mechanical blocking.<br>Electricity heats up the motor assembly up and melting plastic and metal parts.<br>Burn of the pilot possible.	3	7	8	**168**	Temperature sensor is built into the servo. Temperature protection is implemented.	3	7	1	**21**
Structure Parts	Crack due to extreme external force.<br>Hurt of the pilot possible.	4	1	10	**40**					
	Loose shafts or screws.<br>Device falls apart.<br>Hurt of the pilot possible.	3	3	10	**90**	Adhesives are applied to some parts of the device to fix them in position.	3	1	10	**30**
Circuit Board	Failure due to moisture.<br>Device lock down.<br>Lose part of the control of the prosthetic hand.<br>Hurt of the pilot possible.	3	3	10	**90**	Insulation paint is applied to the circuit board.	3	1	10	**30**
Battery	Battery over-heat/over-discharge.<br>Permanent damage to the battery or battery catching fire.<br>Burn of the pilot possible.	5	2	10	**100**	Protection circuit is implemented to the battery to avoid unsafe battery states.	5	1	10	**50**
