# Supplementary figures and images for: HANDSON Hand: Strategies and Approaches for Competitive Success at CYBATHLON 2024
Source: Bioengineering (Basel). 2025 Feb 24;12(3):228. doi: 10.3390/bioengineering12030228 (PMC11939478; doi:10.3390/bioengineering12030228)

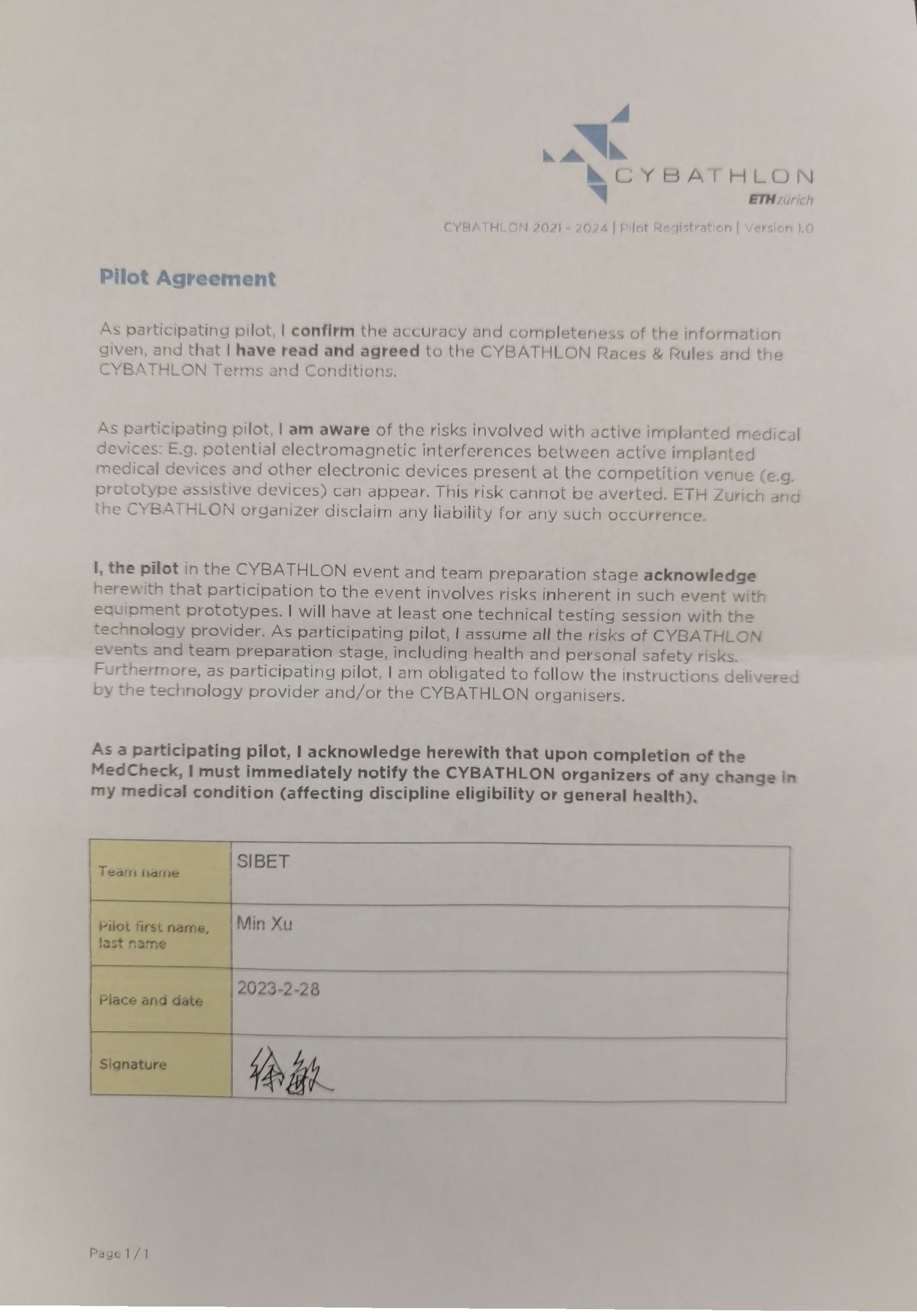

Supplement: Supplementary file 1 [file bioengineering-12-00228-s001.zip › S1. Pilot_Agreement.jpg]
